# Effect of Dietary Hazelnut Peels on the Contents of Fatty Acids, Cholesterol, Tocopherols, and on the Shelf-Life of Ripened Ewe Cheese

**DOI:** 10.3390/antiox10040538

**Published:** 2021-03-30

**Authors:** Vita Maria Marino, Teresa Rapisarda, Margherita Caccamo, Bernardo Valenti, Alessandro Priolo, Giuseppe Luciano, Antonio Natalello, Adriana Campione, Mariano Pauselli

**Affiliations:** 1 Consorzio per la Ricerca nel settore della Filiera Lattiero-Casearia e dell’agroalimentare (CoRFiLaC), S.P. 25 km 5 Ragusa Mare, 97100 Ragusa, Italy; v.marino@corfilac.it (V.M.M.); rapisarda@corfilac.it (T.R.); caccamo@corfilac.it (M.C.); 2 Dipartimento di Scienze Agrarie, Alimentari ed Ambientali, Università degli Studi di Perugia, Borgo XX Giugno 74, 06123 Perugia, Italy; bernardo.valenti@unipg.it (B.V.); adriana.campione@studenti.unipg.it (A.C.); 3 Dipartimento di Agricoltura, Alimentazione e Ambiente, Università degli Studi di Catania, Via Valdisavoia 5, 95123 Catania, Italy; a.priolo@unict.it (A.P.); giuseppe.luciano@unict.it (G.L.); mariano.pauselli@unipg.it (M.P.)

**Keywords:** cholesterol, ewe cheese, fatty acids, hazelnut peels, tocopherols

## Abstract

Hazelnut peel (HNP), a by-product from the chocolate industry, is considered to be a suitable ingredient to be included in the diet of ruminants. This study aimed to evaluate the effect of feeding dairy ewes with a diet containing HNP on ripened cheese quality, including fatty acid (FA) profile, cholesterol, and tocopherol content, as well as stability during storage under commercial conditions. In total, 10 experimental cheeses were produced with bulk milk obtained from ewes fed a commercial concentrate (C group; *n* = 5) or a concentrate containing 36% HNP in dry matter (HNP group; *n* = 5). After 40 days of aging, each cheese was sub-sampled into three slices: one was analyzed immediately (C0 and HNP0), and the other two were refrigerated and analyzed after seven days (C7 and HNP7) and 14 days (C14 and HNP14), respectively. Compared to C, HNP cheese had more than twice as many tocopherols and mono-unsaturated FA and respectively 38% and 24% less of cholesterol and saturated FA. Tocopherols and cholesterol levels remained rather stable up to 14 days of storage regardless of the experimental group, suggesting no cholesterol oxidation. Therefore, the inclusion of HNP in ewe diets could be a valid resource to produce cheese with a healthier lipid profile and higher tocopherols content.

## 1. Introduction

Milk and cheese are an important part of a balanced human diet with a positive role when consumed in moderation. Moreover, the demand for high-quality, healthy dairy products is increasing as consumers are increasingly aware of the link between diet and health. Although dairy products contain molecules with a beneficial health role, such as polyunsaturated fatty acids (PUFA), conjugated linoleic acid (CLA), and vitamins [1], on the other hand, they are rich in saturated fatty acids, particularly myristic and palmitic acids and cholesterol, which are considered a risk to human health [2]; therefore, the current focus of the market has been directed toward the search for dairy products with a healthier lipid composition. The importance of animal diet on the nutritional quality of milk and cheese, with particular regard to the lipid fraction, has been largely discussed in cows [3,4], goats [5], and sheep [6]. Most of the studies carried out so far have been especially focused on ovine milk fat enrichment in CLA and omega-3 fatty acids content by dietary lipid supplementation reporting fatty acid composition, volatile profile, and sensory characteristics [7,8].

However, little is known about the effect of these dietary supplements on the content and stability of cholesterol and vitamins in sheep cheese stored under commercial conditions. Najera et al. [9] reported that bulk milk and cheese from sheep fed rapeseed oilcake concentrate showed a healthier lipid profile with higher content of unsaturated fatty acids and tocopherols and slightly lower cholesterol content [9]. Mele et al. [10] reported that the inclusion of extruded linseed in the diet of dairy ewes enriched cheese in CLA and linolenic acid with a concomitant decrease in saturated fatty acids. On the other side, the same author found in cheese from linseed supplementation compared to control a higher content of cholesterol oxides and malondialdehyde, a lipid peroxidation end product.

The use of extracts [11,12] or by-products [13,14,15] rich in polyphenols, such as condensed or hydrolyzable tannins, have been investigated to improve the quality traits of ruminant products. The use of by-products, in particular, would address the global concern of consumers towards scarcity of natural resources for which FAO promotes the principles of 3R (Reduce, Reuse, and Recycle) for sustainable development in all the productive sectors, including livestock production. For the international dairy research sector, the major challenge gets going to adopt strategies for better reuse of some agricultural industry residues for more environmentally friendly and healthy foods [16]. Accordingly, literature has reported that most food-derived by-products are rich in bioactive molecules that can exert positive effects on both animal welfare and product quality [11]. Among these, the perisperm of the hazelnut kernel, also known as hazelnut peel (HNP), is an example of a suitable ingredient to be included in the diet of ruminants [17,18,19]. Differing from most of the other food-derived by-products, HNP has a low moisture content after the hazelnuts roasting phase and therefore does not need to be stabilized. Moreover, it represents a valuable cheap waste material containing a remarkable content of bioactive compounds such as phenolic compounds (including condensed and hydrolyzable tannins) [20], tocopherols [18], and desirable fatty acids [18,19] that may improve the nutritive value and the shelf-life of milk and cheese.

In a preceding paper, Caccamo et al. [21] reported the effect of the inclusion of HNP in the diet of dairy ewes on sensory characteristics and volatile profile of Pecorino cheese. In the present paper, we have hypothesized that including hazelnut peel in the diet of dairy ewes may affect, in ewe milk cheese, the (1) fatty acids (FA) profile, the cholesterol and tocopherols content, and (2) the extent of cholesterol antioxidant protection (DAP). Moreover, this study aimed to evaluate the effects of refrigerated storage up to 14 days on the stability of cheese FA profiles, and cholesterol and tocopherols content.

## 2. Materials and Methods

### 2.1. Animals, Diet, and Cheese Manufacturing

This study was carried out at the Experimental Farm of the Department of Agricultural, Food and Environmental Science of the University of Perugia (Italy). The research activity reported in this paper treated the supplementation fed to animals by including either beet pulp or hazelnut peel in the concentrate. Therefore, this project is not regulated by the Directive 2010/63/EU article 1, point 4, letter f, on the protection of animals used for scientific purposes, according to which the directive does not apply to the practices not likely to cause pain, suffering, distress or prolonged damage equivalent or superior to that caused by the insertion of a needle according to the good veterinary practices. The feeding trial followed the ordinary practices of dairy sheep farms. Therefore, approval was not needed according to institutional and national guidelines. Details on animals, diet, and cheese manufacturing are available in Campione et al. [17] and Caccamo et al. [21]. Briefly, twenty multiparous Comisana lactating ewes with similar days in milk (89 ± 10 days) and production (827 ± 236 g) levels were equally divided into control (C) and hazelnut peel (HNP) groups. After an adaptation period to the experimental diets, each ewe received chopped alfalfa hay ad libitum and 800g/ewe/day of a pelleted concentrate containing 370 g/kg DM of dried beet pulp (C group) or 360 g/kg DM hazelnut peels (HNP group). Table 1 reports the ingredients and the chemical composition of the dietary treatments.

Bulk milk from each of the two experimental groups was collected during the last week of the experimental period and stored at −30 °C until the quantity of 40 kg was reached. Five batches of cheese were produced with the bulk milk obtained from the respective feeding group, as detailed in Caccamo et al. [21]. After heating the milk at 39 °C, a mixed-strain starter culture (MW039S, Sacco System, CO, Italy) was added, and the mixture was incubated for 10 min. Then, a liquid calf-lamb rennet was added, and after 20 min the curd was turned on the surface and broken into 3 cm large squares to let the whey bleed. After, the curd was further broken to corn grain dimension and put in a plastic basket to remove whey until the pH reached 5.5 level. After brining for 12 h, all the cheese samples were ripened in a cold room for 40 days. Each ripened cheese was sliced into 3 subsamples. One subsample was destined to the analyses without storage (C0 and HNP0), while the other two subsamples were individually wrapped using a PVC (polyvinyl chloride) film, simulating the method adopted in the stores for pre-wrapped products, and used for analyses after 7 (C7 and HNP7) or 14 days of storage (C14 and HNP14) at 8 °C and 80% relative humidity.

### 2.2. Analytical Procedures

Cheese samples were analyzed to determine moisture according to the method proposed by Bradley and Vanderwarn [22], lipid content according to the Gerber-Van Gulik method (ISO 1975), and protein content (total nitrogen × 6.38) determined using the Kjeldhal method.

#### 2.2.1. Determination of Fatty Acid Composition

Cheese fatty acid composition was determined by gas chromatography. Fat was extracted from 5 g of finely minced cheese using a mixture of chloroform and methanol (2:1, *v/v*) as described by Folch et al. [23], and 30 mg of lipids were converted to FA methyl esters (FAME) by base-catalyzed transesterification using 0.5 mL of sodium methoxide in methanol 0.5 N and 1 mL of hexane containing 19:0 as an internal standard. Gas chromatographic analysis was performed on a Trace Thermo Finnigan GC system (ThermoQuest, Milan, Italy) equipped with a flame-ionization detector and a 100 m fused silica capillary column (0.25 mm internal diameter, 0.25 µm film thickness; SP-24056; Supelco Inc., Bellefonte, PA, USA) and helium as the carrier gas (1 mL/min). FAME profile in a 1 µL sample volume (split ratio 1:80) was determined according to the temperature gradient program described by Valenti et al. [24]. The oven temperature was programmed at 50 °C and held for 4min, then increased to 120 °C at 10 °C/min, held for 1 min, then increased up to 180 °C at 5 °C/min, held for 18 min, then increased up to 200 °C at 2 °C/min for 15 min, and then increased up to 230 °C at 2 °C/min, and held for 19 min. The injector and detector temperatures were at 270 and 300 °C, respectively. FAME identification was based on a commercial mixture of standard FAME (Nu-Chek Prep Inc., Elysian, MN, USA), individual standard FAME (Larodan Fine Chemicals, Malmo, Sweden). Fatty acids were expressed as a percentage of total fatty acids. The atherogenicity (AI) and thrombogenicity (TI) indices were calculated according to the following equations:

AI = [(4×C14:0) + C16:0 + C18:0]/[ΣMUFA + ΣPUFAn-6 + ΣPUFAn-3]

IT = (C14:0 + C16:0 + C18:0)/(0.5ΣMUFA + 0.5ΣPUFAn-6 + 3ΣPUFAn-3 + ΣPUFAn-3/ΣPUFAn-6)

#### 2.2.2. Determination of Tocopherols and Total Cholesterol and DAP calculation

The contents of fat-soluble vitamins in relation to cholesterol were determined. In ewe milk, α-tocopherol represents the only major antioxidant inhibiting lipid peroxidation because β-carotene is absent. In addition to the α-tocopherol levels, the contents of cholesterol representing the main target of lipid oxidation [25] were measured. The determination of cheese α-tocopherol and cholesterol was as described by Marino et al. [26] and Oh et al. [27], respectively. Both α-tocopherol and cholesterol were determined by an HPLC method using an SB-C18 column (5-μm particle size, 4.6 nm i.d. × 250 nm, Agilent Zorbax, Agilent Technologies, Santa Clara, CA, USA). The HPLC system (Waters 2695; Waters, Milford, MA, USA) was equipped with a multi-wavelength (λ) fluorescence detector (Waters 2475) using an excitation wavelength of 297 nm and an emission wavelength of 340 nm for the detection of α-tocopherol, equipped with a dual λ absorbance detector (Waters 2487) using a wavelength of 203 nm for the detection of cholesterol. The mobile phases were methanol 100% *v/v* and acetonitrile/methanol/2- propanol (7:3:1, *v/v/v*) for α-tocopherol and cholesterol, respectively. All reagents used were HPLC-grade with a proven purity between 95% and 99.9% and were obtained by Sigma-Aldrich (Sigma Chemical Co., St. Louis, MO, USA). Identification and quantification of α-tocopherol and cholesterol were based on external standards obtained from Sigma (Sigma Chemical Co., St. Louis, MO, USA) with purity ≥ 99.6%. All chemical analyses were done in duplicate.

The degree of antioxidant protection (DAP), used to evaluate the antioxidant protection of foods [25], was calculated as the molar ratio between tocopherols and cholesterol contents in cheese.

### 2.3. Statistical Analyses

Data on cheese chemical composition were analyzed using a one-way ANOVA to test the effect of the dietary treatment (C vs. HNP groups). Data on fatty acid composition were analyzed using a mixed model to test the effect of the dietary treatment (C vs. HNP) and of the days of storage (0, 7, and 14) and their interaction, while the variable batch was considered a random effect (JMP Sas Institute v.12.0.1; Cary, North Carolina, USA). Differences were considered significant at *p* < 0.05. All data are presented as least squares means and standard error of the mean (SEM).

## 3. Results

### 3.1. Diet Effect on Cheese Chemical Composition and Fatty Acid Profile

Fat was higher in cheeses from HNP milk compared with those from C milk (Table 2).

In particular, dietary HNP increased fat content by 21% in cheese compared to the C group.

Dietary supplementation with oleic acid-rich hazelnuts peels resulted in considerable changes in the proportions of the fatty acids categories as well as of individual fatty acids in ewe cheese. Table 3 shows the fatty acid composition of ewe cheeses of both feeding groups.

Specifically, mono-unsaturated fatty acids (MUFA) increased by 114% in HNP compared to the C cheese group, mainly due to the contribution of cis-9 C18:1 that significantly increased by 140%. In contrast, saturated fatty acids (SFA) decreased by 24% in HNP cheeses compared to C. In particular, except for C4:0 and C18:0, all SFA ranging from C6:0 to C16:0 decreased in HNP cheeses compared to C (Table 3).

Moreover, the inclusion of hazelnut peels in the ewe diet was also effective in increasing by 92% C18:2 c9t11 (CLA) in HNP cheeses compared to C. However, in HNP cheeses compared to C, polyunsaturated fatty acids (PUFA) slightly decreased by 5%, and specifically, n-3 fatty acids decreased by 40%, whereas n-6 fatty acids remained unmodified. The addition of hazelnut peels improved the health lipid indices such as atherogenic (AI) and thrombogenic (TI) indices (Table 3).

Finally, a decrease in most odd- and branched-chain (OBCFA) fatty acids were also observed in HNP cheeses compared to the C group (Table 3).

### 3.2. Diet Effect on Fat-Soluble Vitamins and Cholesterol

Alpha-tocopherol, γ-tocopherol, and cholesterol contents in ewe cheeses of both feeding groups were measured (Table 3). To evaluate the effects of diet, α-tocopherol, γ-tocopherol, and cholesterol, referred to as fat content, needed to be compared. The diet affected α-tocopherol and γ-tocopherol of cheese fat. Alpha-tocopherol and γ-tocopherol levels were 2.2 and 3.4 times, respectively, higher in HNP cheeses than C (Table 3). Moreover, cholesterol in fat cheese was also affected by diet. In HNP cheeses, the cholesterol content was 38% lower than in the C group.

### 3.3. Shelf-Life Effect on Fatty Acid Profile, Fat-Soluble Vitamins, and Cholesterol

In this trial, fatty acids, cholesterol, and tocopherols in cheeses of both feeding groups even after 7 (C7 and HNP7) or 14 days of storage (C14 and HNP14) at 8 °C were measured.

Except for α-tocopherol, γ-tocopherol, and cholesterol, cheese FA composition remained rather stable up to 14 days of storage; only a few of cheese FA resulted significantly influenced by storage (Table 3). In particular, C10:0, C11:0, C12:0, and C14:1 c9, C16:1 c9 were slightly decreased after 14 days of storage, whereas some minor C18:1 isomers, including t6 + 7 + 8, t10, and c9 increased after seven days. Only four fatty acids (C15:0 *anteiso*; C16:0 *iso*; C18:1, t10; C18:1, c11) were affected by D × t (Table 3).

## 4. Discussion

In the present study, we focused on the effect of HNP inclusion in a dairy ewe diet on the quality of ripened cheese. In particular, gross composition, fatty acid profile, cholesterol, and tocopherols were investigated during storage under commercial conditions. All the aspects concerning animal performance, chemical and nutritional composition of the milk have been discussed in detail elsewhere [17].

### 4.1. Diet Effect on Cheese Gross Chemical Composition and Fatty Acid Profile

In this study, HNP cheese showed 21% higher fat content compared to C cheeses. The amount of fat eaten by ewes in the HNP group was higher than the C group, and this was probably the reason that explains a higher milk and cheese fat content. Ashes et al. [28] reported that the type and amount of dietary fat supplementation could affect rumen fermentation, milk fat concentration, and FA composition, and consequently, the chemical composition of cheese. In particular, Cannas and Avondo [29] and Cannas et al. [30] recommend, for optimal dairy ewes rumen function and milk fat synthesis, a level of NDF from 33 to 45 g/100 g of DM and of NFC from 28 to 38 g/100 g of DM. In the HNP diet, the content of both NDF and NFC were within the optimal ranges proposed by Cannas and Avondo [29] and Cannas et al. [30], 35.83 and 33.57 g/100 g DM, respectively. However, in the C diet, the values of NDF and NFC were not within the proposed ranges (30.22 and 45.99 g/100 g DM, respectively).

The increase in MUFA and the decrease in SFA found in HNP cheeses compared to C cheese confirm results found in Gomez-Cortes et al. [31] that showed how a higher dietary intake of fat in general and oleic acid, in particular, had the potential for decreasing saturated fatty acids de novo synthesis and increasing healthful fatty acids. Chilliard and Ferlay [5] explained the decrease in short-chain SFA de novo synthesis in the mammary gland with the inhibition of acetyl-CoA carboxylase in long-chain fatty acids rich-diets. It is known that almost all long-chain fatty acids in milk and cheese, besides diet, come from a combination of different biochemical pathways operating both in the rumen through specific microbial ecosystems and in the mammary gland. Thus, according to Harfoot and Hazlewood [32], increased dietary bioavailability of oleic acid results in an increased ruminal level of stearic acid, which is, in turn, largely desaturated to oleic acid through Δ9-desaturase activity in the mammary gland. On the other hand, Mosley et al. [33] demonstrated the ability of mixed ruminal bacteria to convert oleic acid to a multitude of *trans* positional isomers during the process of oleic acid biohydrogenation. In this study, remarkable increases in HNP cheeses were detected for all minor trans-C18:1 isomers (t5, t6 + 7 + 8, t9, and t10) including vaccenic acid (VA; C18:1 t11; +164%). Vaccenic acid is also an intermediate in the rumen of metabolism pathways of linoleic acid (LA; C18:2 c9c12) and α-linolenic acid (LNA; C18:3 c9c12c15).

The increase in CLA could be partially related to the greater percentage of VA found in the HNP group. In fact, the presence of CLA in milk mainly arises from the activity of the Δ9-desaturase in the mammary gland that converts VA to CLA. In this regard, our results were in contrast with those of other authors [7,16], showing that the inclusion of tannin-rich feeds in ewe diet were not efficient in increasing VA and CLA in milk fat and cheese; however, it should be stressed that the ability of tannins to modulate the metabolism of fatty acids depends on many factors, such as the tannin chemical and structural diversity and the dosage and the interaction with the basal diet [34], and therefore it is not possible to generalize on their effects on the fatty acid composition of ruminant products.

Finally, a decrease in most odd- and branched-chain (OBCFA) fatty acids was also observed in HNP cheeses compared to the C group (Table 3). It is known that they are components of the membranes of ruminal bacteria and have activity against some human cancers [35]. According to several authors [9,36,37], the decrease in OBCFA content in milk and cheese could be explained by the higher dietary intake of unsaturated (UFA), which may inhibit cellulolytic ruminal bacteria.

From a human health point of view, the addition of hazelnuts peels enriched cheese fat from oleic acid, one of the healthier fats in the human diet with hypocholesterolemic properties, and positive effects on reduction in plasma triacylglycerol and plasma pressure. Moreover, HNP supplementation cause a reduction in myristic (C14:0) and palmitic (C16:0) acids in cheese fat, whose high intakes raise total plasma cholesterol and low-density lipoproteins (LDL), which are considered an important risk factor for cardiovascular disease and development of the metabolic syndrome. The improvement of nutritional cheese features through the addition of hazelnuts peel is also confirmed by lower health lipid indices, including atherogenic (AI) and thrombogenic (TI) indices (Table 3). In addition, although the ratio of n-6:n-3 PUFA increased in HNP cheeses compared to C, its value of 3:1 was even so below 4:1, considered effective and necessary for meeting the body’s requirements and for promoting health and longevity.

### 4.2. Diet Effect on Fat-Soluble Vitamins, Cholesterol, and DAP

Tocopherols are important for cheese technology because they delay the oxidation of unsaturated fatty acids and cholesterol by extending the shelf-life of the processed milk. Even small changes in the fat-soluble vitamin content of milk could have important effects on oxidation processes and could interact with the ripening process and storage to influence cheese quality.

Higher contents of α-tocopherol and γ-tocopherol and lower content of cholesterol were found in HNP compared to C cheeses. The highest content of tocopherols found in HNP concentrate (Table 1) could explain a higher content of α-tocopherol and γ-tocopherol in HNP cheese. Di Nunzio [38] and Lucchetti et al. [39] also found hazelnut peel rich in tocopherols. As expected in dairy products, α-tocopherol was the main isomer of vitamin E in both cheese groups, but the diet also affected α- and γ-tocopherol proportions, in favor of γ-isomer in HNP cheeses. These dietary effects on α-tocopherol and cholesterol contents have also been found by other authors in cheese made with milk from ewes fed supplemented diets with oleic-acid- and vitamin-E-rich rapeseed concentrate [9], with oleic-acid-rich olive oil [31], and with extruded linseed [10]. However, in HNP cheese compared to rapeseed cheese by Nájera et al. [9], α-tocopherol content was higher (18.1 vs. 13.3 μg/g of fat, respectively). In contrast, in HNP cheese compared to cheeses from dietary rapeseed concentrate [9] and from olive oil supplementation [31], cholesterol content was lower (2.18 vs. 2.27 and 2.89 mg/g of fat, respectively), whereas it was similar to results reported in Mele et al. [10] in cheeses from ewes fed a concentrate with 30% extruded linseed and in Manuelian et al. [40] in Pecorino cheese. With regard to human health issues, the oxidized forms of cholesterol are considered hazardous [41,42]; thus, in addition to the cholesterol content also its potential oxidative stability was investigated by calculating the Degree of Antioxidant Protection (DAP) as reported by Pizzoferrato et al. [25]. The DAP values were strongly related to diet with the highest values in HNP cheese samples compared to C, 7.6 vs. 2.1, respectively. On the basis of the minimum threshold level of 7 × 10^−3^ for cholesterol oxidative stability suggested by Pizzoferrato et al. [25], the cholesterol of C cheese was more likely to be oxidized. However, it cannot be excluded that oxidation may have occurred, especially during cheese-making and during the first 40 days of ripening.

### 4.3. Shelf-Life Effect on Cheese Fatty Acids Profile, Fat-Soluble Vitamins, and Cholesterol

Shelf-life influenced only a few of cheese FA. Todaro et al. [43] also found Pecorino cheese quite stable even up to 180 days of storage and attributed the slight changes of some FA to the residual microbial activity. On the other hand, tocopherol isomers and cholesterol contents in all cheeses of both feeding groups stored up to 14 days did not change (Table 3). The packaging by PVC film, a reference package commonly used in the stores for pre-wrapped products, could have contributed to good oxidative stability of the cheeses, limiting oxygen exchange between the cheese and its outer atmosphere, as well as water losses during cheese storage. However, it cannot be excluded that storage times considered in this trial may be too short to draw conclusions from the differences between the two groups. Possible expected differences could be emphasized by extending the storage time.

## 5. Conclusions

The inclusion of hazelnuts peels in the concentrate feed formulation of indoor dairy ewes was an excellent alternative for obtaining Pecorino cheese with a higher nutritional value and more benefits to human health. Cheese fat was enriched with bioactive components, such as oleic acid, vaccenic acid, CLA, and vitamin E. On the other hand, HNP cheeses showed a significant reduction in cholesterol together with myristic (C14:0) and palmitic (C16:0) acids. In addition, cheeses of both experimental groups had rather stable fat-soluble vitamins, cholesterol, and partially the FA composition content, after up to 14 days of storage. Lastly, besides generating value in the sphere of sustainability, the functional application of agro-food by-products such as hazelnuts peels in the dairy industry as livestock feed could help to reduce the costs of dairy production.

## Figures and Tables

**Table 1:** Ingredients, chemical composition, fatty acids, and bioactive compounds of diets used in the experiment, including concentrates containing either beet pulp (C) or hazelnut peel (HNP).

Items	Hay	Hazelnut Peel	Experimental Concentrates	
			C	HNP
Ingredients (g/kg DM)				
Hazelnut peel			-	360
Barley			345	330
Wheat bran			98.6	97.0
Soybean meal			141	168
Dried beet pulp			370	-
Molasses			25.4	25.0
Calcium carbonate			5.0	5.0
Sodium bicarbonate			5.0	5.0
Dicalcium phosphate			5.0	5.0
Sodium chloride			5.0	5.0
*Chemical composition (g/kg DM)*				
Crude protein	150	78.6	158	163
Ether extract	15.8	226	16.3	91.5
Neutral Detergent Fiber	528	511	302	358
Acid Detergent Fiber	429	388	135	226
Acid Detergent Lignin	95.7	203	15	75.6
Ash	75.6	24.8	63.9	51.8
Protein fractions				
Non-Protein Nitrogen	39.1	1.8	21.5	8.2
B1	6.9	3.7	5.9	19.0
B2	71.0	18.1	100	73.1
B3	19.3	1.6	24.1	32.7
Acid Detergent Insoluble Nitrogen	13.7	53.3	6.1	29.7
*Fatty acids (g/100 g DM)*				
C14:0	0.01	0.02	0.01	0.01
C16:0	0.19	1.04	0.37	0.66
C18:0	0.04	0.38	0.03	0.17
C18:1 *c*9	0.04	11.10	0.27	4.49
C18:2 *c*9 *c*12	0.00	0.18	0.02	0.08
C18:3 *c*9 *c*12 *c*15	0.01	0.02	0.01	0.01
*Bioactive compounds*				
Total phenols (mg/g DM)	7.02	132	2.41	48.2
Total tannins (mg/g DM)	1.32	76.7	0.56	24.6
α-tocopherol (μg/g DM)	9.79	150	3.21	69.9
γ-tocopherol (μg/g DM)	1.10	107	2.62	53.5
δ-tocopherol (μg/g DM)	0.42	7.35	4.57	7.91

C, control diet; HNP, hazelnuts peel diet; DM, dry matter; B1, fast rumen protein degradation, represented by albumin and globulin; B2, intermediate rumen protein degradation, albumins, and glutelins; B3, slow rumen protein degradation, prolamins, extensins, and denatured proteins.

**Table 2:** Chemical composition of cheeses produced with milk of ewes fed with Control (C) or Hazelnut Peels (HNP) diets.

Parameter	Diet		SEM	*p*-Value
	C	HNP		
Chemical composition (g/kg dry matter)				
Moisture	40.1	38.9	1.551	NS
Total solids	61.2	64.2	0.260	***
Fat	26.5	31.9	0.817	***
Ash	3.76	3.29	0.089	***
Protein	25.3	24.3	1.287	NS

NS, not significant; ***, *p* < 0.001; SEM: standard error of the mean.

**Table 3:** Fatty acid profile, tocopherols, Degree Antioxidant Protection (DAP), and nutritional indices of cheese from ewes fed control and hazelnuts peel diets.

Item	Diet		SEM	*p*-Value		
	C	HNP		Diet (D)	t[v1] ime (t)	D × t
Fatty acid or groups of FA (g 100g^−1^ fatty acids)						
C4:0	2.27	2.60	0.110	*	NS	NS
C6:0	2.61	2.0	0.083	***	NS	NS
C8:0	2.86	1.73	0.059	***	NS	NS
C10:0	9.58	4.80	0.144	***	*	NS
C11:0	0.50	0.24	0.008	***	*	NS
C12:0	5.75	2.74	0.058	***	**	NS
C12:1 *c*9	0.23	0.10	0.003	***	NS	NS
C14:0 *iso*	0.15	0.09	0.002	***	NS	NS
C14:0	12.5	8.40	0.068	***	NS	NS
C14:1 c9	0.27	0.15	0.005	***	*	NS
C15:0 *iso*	0.28	0.19	0.003	***	NS	NS
C15:0 *anteiso*	0.58	0.35	0.007	***	NS	**
C15:0	1.46	0.97	0.009	***	NS	NS
C16:0 *iso*	0.40	0.24	0.003	***	NS	*
C16:0	29.4	20.3	0.196	***	NS	NS
C16:1 *t*9 + C17:0 *iso*	0.35	0.28	0.026	NS	NS	NS
C16:1 *c*7	0.17	0.24	0.012	***	NS	NS
C16:1 *c*9	1.11	0.65	0.006	***	**	NS
C17:0 *anteiso*	0.52	0.39	0.014	***	NS	NS
C17:0	0.60	0.42	0.025	***	NS	NS
C17:1 *c*9	0.30	0.15	0.008	***	NS	NS
C18:0	4.05	10.26	0.186	***	NS	NS
C18:1 *t*5	<0.001	0.05	0.002	***	NS	NS
C18:1 *t*6 + *t*7 + *t*8	0.07	0.53	0.008	***	**	NS
C18:1 *t*9	0.14	0.61	0.013	***	NS	NS
C18:1 *t*10	0.21	0.59	0.021	***	*	*
C18:1 *t*11	0.63	1.66	0.029	***	NS	NS
C18:1 *c*6	0.47	0.76	0.017	***	NS	NS
C18:1 *c*9	11.4	27.3	0.331	***	NS	NS
C18:1 *c*11	0.42	0.72	0.010	***	*	*
C18:1 *c*12	0.24	0.41	0.131	NS	NS	NS
C18:1 *c*13	0.03	0.13	0.042	NS	NS	NS
C18:1 *c*14	0.18	0.36	0.040	*	NS	NS
C18:2 *t*8*c*13	0.28	0.37	0.052	NS	NS	NS
C18:2 *t*9*c*12	0.26	0.37	0.043	NS	NS	NS
C18:2 *t*9*c*13	0.12	0.09	0.009	*	NS	NS
C18:2 *c*9 *t*11	0.41	0.79	0.027	***	NS	NS
C18:2 *c*9*c*12	3.49	3.51	0.040	NS	NS	NS
C20:0	0.16	0.17	0.008	NS	NS	NS
C18:3 *c*6*c*9*c*12	0.10	0.02	0.003	***	NS	NS
C18:3 *c*9*c*12*c*15	1.64	1.02	0.020	***	NS	NS
C20:1*c*11	0.05	0.05	0.009	NS	NS	NS
C20:4 *c*5*c*8*c*11*c*14	0.15	0.11	0.007	*	NS	NS
C20:5 *c*5*c*8*c*11*c*14*c*17	0.09	0.03	0.004	***	NS	NS
C22:0	0.07	0.06	0.005	NS	NS	NS
C 22:5 *c*7*c*10*c*13*c*16*c*19	0.23	0.09	0.047	NS	NS	NS
C22:6 *c*4*c*7*c*10*c*13*c*16*c*19	0.04	0.06	0.010	NS	NS	NS
C23:0	0.06	0.05	0.006	*	NS	NS
C20:3 *c*8*c*11*c*14	0.004	0.003	0.002	NS	NS	NS
C24:0	0.03	0.06	0.012	NS	NS	NS
Σn-3	2.00	1.19	0.052	***	NS	NS
Σn-6	3.74	3.65	0.043	NS	NS	NS
Σn-6/Σn-3	1.89	3.06	0.050	***	NS	NS
SFA	73.8	56.1	0.369	***	NS	NS
MUFA	16.2	34.8	0.261	***	NS	NS
PUFA	6.81	6.45	0.092	*	NS	NS
Σn-9	11.4	27.4	0.334	***	NS	NS
MUFA/SFA	0.22	0.62	0.006	***	NS	NS
PUFA/SFA	0.09	0.12	0.002	***	NS	NS
AI	63.3	41.8	0.337	***	NS	NS
TI	14.1	7.97	0.184	***	NS	NS
HH	45.9	52.2	0.547	***	NS	NS
Tocopherols (µg 100 g^−1^ cheese)						
α-Tocopherol	215	578	13.533	***	NS	NS
γ-Tocopherol	32.8	135	0.210	***	NS	NS
Total cholesterol (mg 100g^−1^ cheese)	92.7	69.5	2.149	***	NS	NS
α-Tocopherol/γ-Tocopherol	6.13	3.83	0.338	***	NS	NS
Tocopherols (µg g^−1^ fat)						
α-Tocopherol	8.10	18.1	0.451	***	NS	NS
γ-Tocopherol	1.25	4.23	0.123	***	NS	NS
Total cholesterol (mg g^−1^ fat)	3.50	2.18	0.078	***	NS	NS
DAP (10^−3^)	2.09	7.62	0.159	***	NS	NS

Abbreviations are: C, control diet; HNP, hazelnuts peels diet; t, shelf-life time. Σn-6: (C18:2 c9c12 + t9c12 + C18:3 *c*6*c*9*c*12 + C20:4 *c*5*c*8*c*11*c*14); Σn-3: (C18:3 c9c12c15 + C20:5 *c*5*c*8*c*11*c*14*c*17 + C22:5 *c*7*c*10*c*13*c*16*c*19 + C22:6 *c*4*c*7*c*10*c*13*c*16*c*19); AI (Atherogenicity Index) = [(C12:0 + (4 × C14:0) + C16:0)/(ΣMUFA) + Σω6 + Σω3); TI (Thrombogenicity Index) = (C14:0 + C16:0 + C18:0)/[(ΣMUFA × 0.5) + (0.5 × Σω6) + (3 × Σω3) + (Σω3/Σω6)]; HH, Hypocholesterolemic fatty acids (C18:1 c9 + C18:2 n6 + C18:3 n3 + C20:5 n3 + C22:6 n3)/Hypercholesterolemic fatty acids (C14:0 + C16:0); DAP, Degree Antioxidant Protection; SFA, saturated fatty acids; MUFA, monounsaturated fatty acids; PUFA, polyunsaturated fatty acids. Values are Least squares means (D: *n* = 10; t: *n* = 30). Variables values within a row with different superscripts differ significantly. Significance of effects indicated by: * *p* < 0.05; ** *p* < 0.01; *** *p* < 0.001; NS, not significant (*p* > 0.05).

## Data Availability

The data presented in this study are available on request from the corresponding author.
